# Stakeholders’ Perceptions of Agronomic Iodine Biofortification: A SWOT-AHP Analysis in Northern Uganda

**DOI:** 10.3390/nu10040407

**Published:** 2018-03-24

**Authors:** Solomon Olum, Xavier Gellynck, Collins Okello, Dominic Webale, Walter Odongo, Duncan Ongeng, Hans De Steur

**Affiliations:** 1 Department of Agricultural Economics, Ghent University, Coupure Links 653, 9000 Ghent, Belgium; solomon.olum@ugent.be (S.O.); Xavier.Gellynck@UGent.be (X.G.); odongo78@gmail.com (W.O.); 2 Department of Food Science and Postharvest Technology, Gulu University, P.O. Box 166, Gulu, Uganda; duncanongeng@hotmail.com; 3 Department of Biosystems Engineering, Gulu University, P.O. Box 166, Gulu, Uganda; collins.okello@gmail.com; 4 Department of Rural Development and Agribusiness, Gulu University, P.O. Box 166, Gulu, Uganda; webale2009@yahoo.com; 5 School of Agricultural and Environmental Sciences, Mountains of the Moon University, Fort Portal, Uganda

**Keywords:** agronomic biofortification, iodine deficiency, stakeholder analysis, analytical hierarchy process, SWOT analysis, Uganda

## Abstract

Agronomic biofortification (i.e., the application of fertilizer to elevate micronutrient concentrations in staple crops) is a recent strategy recommended for controlling Iodine Deficiency Disorders (IDDs). However, its success inevitably depends on stakeholders’ appreciation and acceptance of it. By taking Northern Uganda as a case, this study aimed to capture and compare the perceptions of seven key stakeholder groups with respect to agronomic iodine biofortification. Therefore, we employed a SWOT (Strength, Weaknesses, Opportunities & Threats) analysis in combination with an Analytical Hierarchy Process (AHP). Findings show that stakeholders (*n* = 56) are generally positive about agronomic iodine biofortification in Uganda, as its strengths and opportunities outweighed weaknesses and threats. Cultural acceptance and effectiveness are considered the most important strengths while the high IDD prevalence rate and the availability of iodine deficient soils are key opportunities for further developing agronomic iodine biofortification. Environmental concerns about synthetic fertilizers as well as the time needed to supply iodine were considered crucial weaknesses. The limited use of fertilizer in Uganda was the main threat. While this study provides insight into important issues and priorities for iodine biofortification technology in Uganda, including differences in stakeholder views, the application of the SWOT-AHP method will guide future researchers and health planners conducting stakeholder analysis in similar domains.

## 1. Introduction

Agriculture can inevitably play a big role in improving nutrition and health in developing countries. However, advancement in agriculture has often concentrated on increasing production to avert food insecurity, at the expense of improving the nutrient content of the food crop [1]. As such, many farming systems currently cannot produce enough micronutrients to meet human requirements in a sustainable way [2]*.* Unfortunately, the staple foods that dominate the diets of most people in the developing part of the world have low amounts of micronutrients, such as iodine, iron, vitamin A, zinc and selenium [3,4]. This has resulted in high prevalence rates of micronutrient malnutrition, commonly called hidden hunger. It is estimated that almost half of the world’s population are suffering from one or more micronutrient deficiencies, and the number is on the rise [3,5]. Iodine deficiency alone affects about 2 billion people, most of whom live in developing countries [6,7]. In fact, nearly 35 percent of the global population are estimated to have an inadequate iodine intake [8]. Iodine is needed in humans for the production of thyroid hormones, and its deficiency presents a number of poor health outcomes, collectively termed as Iodine Deficiency Disorders (IDDs). The most common manifestations of IDDs include the enlargement of thyroid gland (goiter), preventable mental retardation and cretinism [6,9]. 

Iodine is naturally abundant in fish and other sea foods, which are not regularly consumed by resource poor households and those living inland, in mountainous areas and in regions with frequent floods, i.e., those far from oceans, where soils lack sufficient iodine for plant uptake [9,10]. Universal Salt Iodization (USI) has been the preferred strategy for increasing iodine intake to eliminate IDDs [11]. Despite worldwide promotion and consumption of iodized salt, IDDs is still a widespread set of diseases of public health significance [11], which raises the question of whether USI (alone) is the most appropriate strategy for combating IDDs in various regions. Furthermore, high salt consumption is being discouraged on account of its association with the increased occurrence of cardiovascular diseases, such as hypertension [12]. As health practitioners advocate for a reduction in the quantity of salt consumed due to health risks associated with it, the risk and prevalence of IDDs will remain high. This situation has prompted the World Health Organization to recommend finding alternative approaches for improving iodine intake [8]. 

Biofortification, the process of enriching staple foods with essential micronutrients, is one of the key alternatives recommended to increase iodine availability and consumption. Within the wider realm of the biofortification strategy is agronomic biofortification, which can be achieved through application of micronutrient-rich fertilizers to specific crops of interest. Recent empirical studies have provided results demonstrating the potential of agronomic biofortification for enriching crops with iodine (for a review, see [7]). While, it can only be used to enrich staple crops with mineral elements (e.g., iodine) and not organic nutrients (e.g., vitamins), agronomic biofortification is a relatively simple and fast strategy [3]. However, as is the case with any other technological interventions at the community level, the success of agronomic iodine biofortification heavily depends on stakeholders’ appreciation and acceptance. Stakeholders are important because they are suppliers, customers, implementers or regulators of such an intervention and they make tradeoffs between tangible and intangible benefits and risks when making decisions regarding an intervention [13]. Therefore, there is a need to understand stakeholders’ perceptions of acceptance and future adoption of agronomic iodine biofortification.

The currently existing stakeholder literature on biofortification has mainly concentrated on consumers (see reviews of Birol et al.; De Steur et al.; and Talsma et al. [14,15,16]). These reviews synthesized studies on consumers’ evaluation of biofortified foods by applying sensory evaluation, hedonic trait analyses or economic valuation methods (i.e., willingness-to-pay). Some of them have applied stakeholders’ rational decision-making theories to understand the contribution of both extrinsic (e.g., technological features) and intrinsic (e.g., attitude) factors. For example, De Steur et al. [17] applied the Protection Motivation Theory (PMT) to understand the parents’ and school heads’ intentions to include iodine biofortified foods in school meals, while the study of Talsma*,* et al. [18] applied the Theory of Planned Behaviour (TPB) and Health Belief Models (HBM) to evaluate the acceptability of pro-vitamin A biofortified cassava by caretakers of school-going children. Only few studies have examined farmers’ reactions to biofortification or specific biofortified foods. For example, Birol and her colleagues applied choice experiments to investigate farmers’ preferences for consumption and production traits of iron biofortified pearl millet in India [19] and their interest in cultivating Genetically Modified (GM) maize in Mexico [20] (for a review on consumers’ and producers’ acceptance and adoption of biofortified foods, see Talsma et al. [14]). A critical look at all stakeholder studies on biofortification reveals that non-consumer stakeholders have hardly been examined, and only few studies have examined a combination of stakeholders: mostly consumers and farmers. In this study, we aim to analyze the perceptions of multiple stakeholder groups on agronomic iodine biofortification.

There are various tools for stakeholder analysis that could be applied to the field of food, agriculture and the environment, such as Strengths, Weaknesses, Opportunities and Threats (SWOT), Stochastic Multicriteria Acceptability Analysis (SMAA), Stochastic Multicriteria Acceptability Analysis with Ordinal criteria (SMAA-O), Simple Multi-Attribute Rating Technique (SMART), Analytical Hierarchy Process (AHP) (for a review, see [21]), S-O-S (SMAA-O in SWOT) [22], Strategic Orientation Rounds (SOR) [23] and Delphi Rounds [24]. In order to capture perceptions of stakeholders on agronomic iodine biofortification, we have opted for a combination of the SWOT (Strengths, Weakness, Opportunities and Threats) analysis and the Analytical Hierarchy Process (AHP). Whereas the SWOT analysis is a common tool used in strategic marketing management to appraise projects, the qualitative information it generates is often considered inadequate for prioritizing and facilitating decision-making [25]. The integration of the Analytical Hierarchy Process (AHP) overcomes this issue as it applies a quantitative approach to the information generated from SWOT analysis. AHP is one of the most widely applied Multi-Criteria Decision Analysis (MCDA) tools, owing to its ability to analyze contradictory preferences and quantify and compare qualitative views of stakeholders [26]. The novelty of the AHP method is that, unlike in the classical multivariable modelling approaches, which generate utility functions, the AHP tool is based on pairwise comparison of preferences [27], which enables the weighing of each factor against all other factors, one at a time, before determining the overall influence according to the eigenvalue method (priority scoring approach) [28]. It applies a mathematical approach to solve complex problems in decision making [25] and has been applied to a variety of areas, including economics, management and policy/strategy design [29,30,31]. The AHP method has recently gained interest among researchers in the field of agriculture. It was used, for example, to analyze preferences for conservation agriculture [26], to identify differences in farming for the rural–urban interface [32], to explore perceptions of smallholders on agroforestry [33] and to evaluate the adoption of silvopasture [34]. While these studies make important contributions to the application of AHP methodology to agricultural research, they consider a narrow scope of stakeholders, often leaving out either farmers, suppliers, government/policy influencers or researchers. 

The aim of this paper is to conduct a multi-stakeholder SWOT-AHP analysis to identify the most important factors that can inform the development of agronomic iodine biofortification in Uganda. Specifically, this study intends to (1) document stakeholders’ perceived strengths, weaknesses, opportunities and threats for the development of agronomic iodine biofortification; (2) carry out a comparative analysis on the perceptions of different stakeholder groups on agronomic iodine biofortification; and (3) provide recommendations for future implementation of iodine biofortification from a developer’s and policy maker’s point of view. Through accomplishing our objectives above, this study contributes to the growing literature on the application of Analytical Hierarchy Process to appraise agricultural interventions and is considered the first application of the SWOT-AHP method on agriculture–nutrition linkages and micronutrient interventions, in particular. 

The study takes place in Northern Uganda. According to the recent Ugandan demographic and health survey, this region presents some of the worst indicators of malnutrition, including micronutrient deficiency [35]. In addition, the region is dominated by rural, poor smallholders who farm on marginally fertile land, which potentially lacks adequate iodine for crop intake [17,36,37]. The rural poor in this landlocked region further have low consumption of iodine rich foods, such as fish [38,39], which puts them at risk of inadequate iodine intake and IDDs. 

## 2. Materials and Methods

### 2.1. Stakeholder Sample

A SWOT-AHP analysis was conducted using a workshop procedure with representatives of seven key stakeholder groups in Gulu municipality (Northern Uganda): (1) councilors (*n* = 7), i.e., government officials from district councils involved in local government policy formulation; (2) academia (*n* = 9), i.e., researchers working in a higher education institution setting and agriculture, food and nutrition departments; (3) government extension officers (*n* = 12), consisting of both District Agricultural Officers (DAOs) and agricultural officers attached to 5 districts in Northern Uganda (Gulu, Lira, Kitgum, Agago and Otuke); (4) NGO (Non-Governmental Organization) extension officers (*n* = 8) from international NGOs active in Northern Uganda implementing nutrition or agriculture/livelihood programs in the region; (5) CDO groups (*n* = 8), consisting of Community Development Officers (CDOs) and district nutrition focal persons from Northern Uganda; (6) agro-input representatives (*n* = 5), consisting of representatives from seed companies and agro-input shops who sell and buy planting materials (e.g., seeds); and (7) elite farmers (*n* = 7), who are educated farmers operating either commercially or through subsistent farming in Northern Uganda. 

In total, there were 56 participants, with at least 5 representatives from each stakeholder group—a sufficient number to obtain expert opinions on the topic. The SWOT-AHP method can be implemented with small or large samples as it does not require deriving a confidence interval around the mean and making inferences to the population from which the samples are drawn [34]. The diversity in the backgrounds of these stakeholder groups allowed us to identify variability in SWOT factors and scores for implementing agronomic iodine biofortification. 

### 2.2. SWOT Method

The SWOT analysis has been widely used to formulate strategies for new technologies or interventions and prioritize between various existing options [40,41,42]. It involves a process of thorough thought and identification of factors related to, for example, a new or existing technology or product, in order to enable its development or improvement. It is the most common tool used to analyze internal and external factors during project strategy development phases [23,42,43]. Here, the internal factors consist of the strengths (positive) and weaknesses (negative) of agronomic iodine biofortification, while the external factors are the opportunities (positive) and threats (negative) that exist for the implementation of this intervention. An effective strategy is one that maximizes strengths and opportunities while minimizing weaknesses and threats [43]. As it is advisable to execute SWOT analysis by people who are familiar with the topic [23]—in this case, the key stakeholders and experts in agriculture, health and nutrition—the tool allowed us to identify important factors that could influence the implementation of agronomic iodine biofortification. 

### 2.3. AHP Method

The first step in applying the AHP method to SWOT analysis involves pairwise comparisons of factors generated within each SWOT category (S,W,O,T). The comparisons are made separately for all factors within a SWOT category, and a priority value for each factor is computed using the eigenvalue method [30,33]. The comparison is carried out on a scale from 1 to 9, where 1 indicates that the 2 factors of a pair are considered equally important, while 9 means that one factor is of extreme importance relative to the other factor [28,29]. Table 1 exemplifies a pairwise comparison set. 

In the second step, SWOT categories are compared to each other using the aforementioned 9-point scale in order to determine the overall influence of factors (“which of the 4 SWOT aspects has the highest importance within the scope of agronomic iodine biofortification”). This helps to determine the scaling factor of each SWOT category.

### 2.4. Data Collection

The data collection was conducted following 3 sessions. First, a series of short presentations (information rounds) was given. Participants were informed about the objectives of the workshop, common micronutrient deficiencies (e.g., iron, vitamin A, zinc and iodine deficiency) and universally applied interventions, such as dietary diversification, food fortification, supplementation and biofortification. As agronomic biofortification is a relatively new approach in modern agriculture, participants were given presentation on the types of biofortification (conventional breeding, genetic modification and agronomic biofortification) [1]. The reason for discussing biofortification and other micronutrient approaches was to reduce a positive bias of participants towards agronomic biofortification. At the end of the workshop, more details were revealed with respect to the objective of the study, and feedback was obtained. 

In a second workshop session, participants were assigned to small groups that consisted of 3–5 members from different stakeholder groups. Thereby, participants were asked to brainstorm on the SWOT factors of agronomic iodine biofortification. Before this exercise, a short presentation was given on how to participate in SWOT analysis for evaluating an intervention. Based on the outcomes of the pilot test, particular attention was devoted to the division between internal (S, W) and external (O, T) aspects. The group-based SWOT factors were presented, and closely related factors were combined into broader categories, resulting in a final non-limitative SWOT list. Only aspects with consensus among the participants (reached by majority acceptance) were included. Next, the participants ranked their 5 top factors for each SWOT category. These were the factors that were used in the subsequent comparisons. 

In a third session, participants worked again in groups, but were assigned to a group according to their stakeholder category, e.g., all participants from seed companies formed one group. Participants were asked to conduct pairwise comparisons: first, between the 5 top factors within each category (as identified in session 2) and second, between the SWOT categories (e.g., all strengths together versus all opportunities together). During this session, stakeholders were told to critically assess the factors one pair at a time and honestly quantify their relative importance. They were discouraged to guess as this leads to inconsistent and irrelevant results. 

### 2.5. Data Analysis

Microsoft Excel 2016 was used to estimate priorities based on the pairwise comparisons for each stakeholder group and to check for the consistencies. 

To estimate priorities, the results of the pairwise comparisons can be represented in a reciprocal matrix with the relative weight (*w*) represented by *a_ij_* (where *a_ij_* is the element of row *i* and column *j*) and its reciprocal 1/*a_ij_* (on the opposite side of the diagonal):

$$A=aij=\left[\begin{array}{c} w1w1\;\;w1w2\;\;w1w3\ldots .w1wn\\ w2w1\;\;w2w2\;\;w2w3\ldots w2wn\\ w3w1\;\;w3w2\;w3w3\ldots .w3wn\\ \;\;\;\;\vdots \;\;\;\;\;\;\;\;\;\;\;\;\vdots \;\;\;\;\;\;\;\;\;\;\;\vdots \;\;\;\;\;\;\;\ldots .\;\;\;\;\;\;\vdots \;\;\;\;\;\\ wnw1\;\;wnw2\;\;wnw3\ldots wnwn \end{array}\right]$$

To obtain normalized relative weights, the pairwise reciprocal matrix A is normalized by dividing each element of the matrix with the total of its column. For each factor, two priority scores (eigenvalues) can be developed: local or global priority scores. While the former measures priorities within each SWOT category, the later prioritizes across categories. The local priority scores for SWOT factors, also known as scaling factor scores of the SWOT categories, are determined by summing elements in each row of the normalized matrix and dividing by the number of elements in the row. The global priority score of each factor is determined by multiplying its priority score with the scaling factor of its SWOT group. Matrix A is governed by the rule that *a_ij_* > 0, and when *i* = *j*, *a_ij_* = 1 [30], and the sum of all scores in the normalized matrix equals one. As such, priority scores can be interpreted in terms of individual values, e.g., 0.2, and as percentages (0.2 = 20%), in line with previous studies [30,33].

During pairwise comparisons, inconsistencies often occur due to the subjective nature of human judgement [33]. Checking consistency is a key and inherent step of AHP analysis, to obtain more reliable results [28,29]. Therefore, it is required that when inconsistency is detected, the values in the related matrices are re-examined to obtain the required consistency [28]. Matrix A was examined for inconsistencies using the following formula:

$$\mathrm{CI}=\frac{{ʎ}_{\text{max}}\;–\;n}{n\;–\;1\;}\;\text{CR}=\text{CI}/\text{RI}$$

where CR is the consistency ratio, CI is the consistency index, RI is a random index produced by a random matrix of order *n* (see Saaty [29] for a random index table), *n* is the number of pairwise comparisons and ʎ_max_ is the largest eigenvalue. 

The largest eigenvalue is equal to the number of comparisons (ʎ_max_ = *n*). Inconsistency occurs when ʎ_max_ deviates from *n*, as a result of inconsistent responses in pair-wise comparisons [44]. The likelihood of getting inconsistent responses increases when the number of elements to be compared is larger. The general rule states that CR (CI/RI) should not exceed 0.1 (10%) [28]. Data from the first round of pairwise comparisons were checked for consistency, in line with previous studies [30,31,33], and only a small group of participants was contacted after the workshop to make minor adjustments so that CR is at or below the recommended 10% threshold. 

## 3. Results and Discussion

### 3.1. Stakeholders’ Key Factors Influencing Agronomic Iodine Biofortification (SWOT Analysis)

Table 2 depicts the positive (strengths and opportunities) and negative (weakness and threats) factors for implementing agronomic iodine biofortification in Northern Uganda. The key strength, as agreed upon by stakeholders, is that agronomic iodine biofortification is a relatively inexpensive approach to prevent IDDs. They consider the technology to be relatively simple and applicable to farmers in a Ugandan setting, on top of being culturally acceptable. It is further perceived that it can be effective in reducing chronic disease risks and it can address food security and health concerns simultaneously. Worth noting among the key opportunities include the existence of government support and extension service structures for the promotion of modern agriculture, a supportive fertilizer policy in Uganda, high prevalence of IDDs with visible goiter cases seen in the community and availability of abundant iodine deficient land for agricultural production in Northern Uganda. 

With respect to perceived weaknesses of the technology, stakeholders pointed out that fertilizers are expensive and often not affordable to many farmers, unless subsidized. They are also worried about the environmental impact of the over-use of fertilizers and that agronomic biofortification takes a long time to supply iodine as one has to wait until the crops are mature. The participants also expressed concern over the lack of readily available iodine fertilizers in the country. They further raised concerns over the fact that iodine is volatile and can easily be lost from the soil or plants after application. Some of the threats generated included the following: fertilizer application is affected by environmental factors, e.g., soil moisture and pH, general use of fertilizers by farmers in Uganda is low, there are competing needs in regard to increasing yield versus quality (nutrients) of food crops, and there have been increasing campaigns in the country to lower the use of synthetic fertilizers in favor of organic fertilizers. One interesting outcome of the SWOT analysis was the envisaged threat that there would be a misconception of agronomic biofortified foods as genetically modified organisms. Participants stated this is a threat, especially given that there is no visible color change in foods biofortified with iodine. 

### 3.2. Comparison of Stakeholder Perceptions of Agronomic Iodine Biofortification (AHP Analysis) 

By applying the quantitative AHP method to the SWOT output, stakeholder groups scored differently on the importance of each of the identified factors, as shown through the priority values (Table 3). The local priority scores reflect the relative importance of each factor within a SWOT category, while the global priority scores demonstrate the relative importance of each factor across all SWOT categories. It is important to note that the priority scores of the factors are relative values originating from pairwise comparisons made by the stakeholders. In other words, factors with low priority values are less important, rather than not important, for successful implementation of agronomic iodine biofortification. Data from the councilor group were excluded as consensus could not be reached during pairwise comparison, and a high degree of inconsistency in responses was reported. In all their comparisons, the consistency ratio was above the recommended 0.1 threshold. 

**Figure 1:**
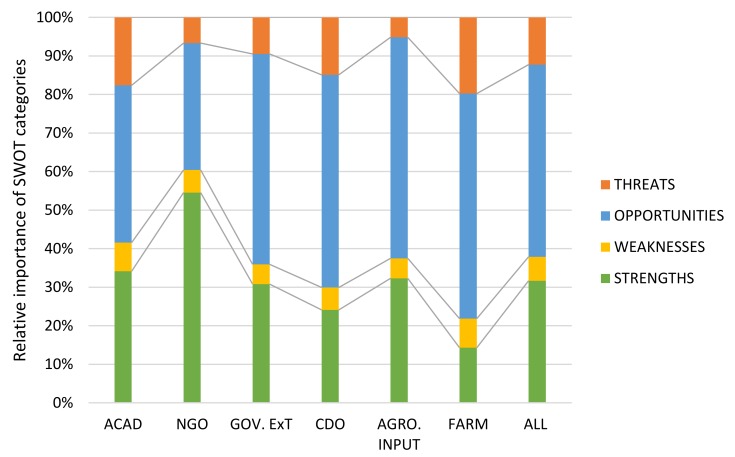
Stakeholder group perceptions of the importance of SWOT categories for the development of agronomic iodine biofortification in Uganda. ACAD: academic group; GOV.EXT: government extension group; CDO: Community Development Officers; AGRO.INPUT: agro-input company sample; FARM: elite farmers group; NGO, Non-Governmental Organization.

The SWOT-AHP analysis shows that, on average, the consulted stakeholders hold a generally positive perception about agronomic iodine biofortification (Table 3). Therefore, the opportunity category is the most important, with an average priority score of 0.499 (49.9%). The average priority score for all strengths combined was 0.317 (31.7%) while threats and weaknesses only obtained scores of 0.123 (12.3%) and 0.062 (6.2%), respectively. Figure 1 visualizes the differences among stakeholder groups in terms of the relative importance of SWOT categories, e.g., strengths versus weaknesses. The results show that for nearly all stakeholder groups, the opportunities category was considered to contain the most important factors for the implementation of agronomic iodine biofortification in Northern Uganda. Only the NGO group put the strength category forward as being the most important. In addition, the academic and farmers groups found the threat category relatively more important than other stakeholder groups. 

The importance of SWOT factors according to stakeholder groups are also presented in Table 3 (graphical perception maps of the priority values for each stakeholder group are available in Supplementary Figures S1–S6). In these maps, the line in each quadrant shows the cumulative weight of factors in each SWOT category, and the length of the line shows the relative importance of a SWOT category compared to the other categories. 

#### 3.2.1. Strengths

**Figure 2:**
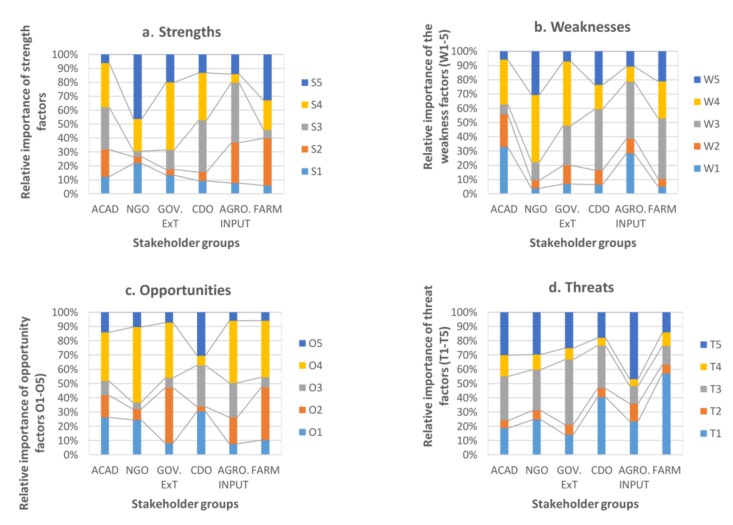
Perceptions of stakeholders on importance of (**a**) strengths; (**b**) weaknesses; (**c**) opportunities and (**d**) threats of agronomic iodine biofortification. ACAD: academic group; NGO, Non-Governmental Organization; GOV.EXT: government extension group; CDO: Community Development Officers; AGRO.INPUT: agro-input company sample; FARM: elite farmers group. Note: For a list of descriptions of each factor, see Table 2 and Table 3.

Analysis of within-SWOT categories for strengths shows that effectiveness (S4: *effective in reducing chronic disease risk*) and the potential for multi-biofortification (S5: *iodine fertilizers can be blended with other plant nutrients*) are the most important strengths of agronomic iodine biofortification (Figure 2a). There has been huge progress in reducing IDDs in Uganda since the introduction of iodized salt in 1994 [45], clearly demonstrating the effectiveness of the USI strategy. However, due to the concern that high consumption of salt increases risk for chronic diseases [12], WHO has recommended a reduction in salt intake [46], while also supporting alternative strategies for controlling IDDs [8] and thus, reducing the risk of cardiovascular diseases. The stakeholders consulted in this study perceived that agronomic iodine biofortification could be effective in reducing chronic disease risk (such as hypertension and other cardiovascular diseases). However, this remains to be seen once iodine biofortified foods are disseminated. Stakeholders’ perception that iodine fertilizers have the ability to offer other nutrients was scored highest by NGO representatives and second highest by the farmers’ representatives. The current soil fertility management strategies in Uganda and other East African countries (e.g., recycling plant residues and land fallow) have been found to be inadequate for replenishing soil nutrients [47]. Therefore, the availability of iodine fertilizers that offer other plant essential nutrients would be beneficial to the farmers. Recent studies that have demonstrated the ability of agronomic biofortification to enrich crops with iodine have applied iodine in the form of potassium iodide (KI) and potassium iodate (KI0_3_) [4,48]. While potassium (K) is an essential nutrient for plant growth [49], it is not known if KI or KI0_3_ applied to provide iodine makes K available for plant uptake and growth. Nevertheless, potassium fertilizers have been used in Uganda and many countries to support crop growth. The academic, CDO and agro-input groups gave high scores to strength S3 (*culturally acceptable in Uganda*). Like any other agri-food intervention implemented in the community, culture is a very important factor in the acceptance of biofortification. The success of biofortification depends on whether biofortified staples are cultivated by famers and accepted by consumers in a particular community [15,50]. Culture is particularly important in food-based interventions (such as biofortification) because what is accepted in one culture may be totally rejected in another [51]. The CDO and agro-input companies are directly involved in distributing inputs as well as providing support to the community, which could explain their high priority scores for this aspect. Unlike other stakeholders (Figure 2a), the elite farmers group scored the highest priority to S2 (*simple and easy to implement*). This could be related to the fact that farmers are directly involved in primary production and are therefore more likely to value the ease of implementation of a technology than other stakeholders. Previous research on the Technology Acceptance Model (TAM), for example, shows the importance of perceived ease of use and usefulness as very critical for acceptance of a technology [52,53], including iodine biofortification [54]. As such, the ease of implementing agronomic biofortification is considered important to farmers as it is a precondition for their acceptance and future adoption. 

#### 3.2.2. Opportunities 

In the case of opportunities, all stakeholders generally view the *high prevalence of IDDs in Uganda* (O4) (Figure 2c) as the most important opportunity for developing agronomic iodine biofortification in Uganda. While there has been no recent study on general prevalence of goiter and IDDs in Uganda, Bimenya et al. (2002) [37] reported a total goiter rate of up to 60.2% with 30% visible goiter in school-going children in Uganda. Due to the large investments of the government of Uganda [55] to tackle major micronutrient deficiencies, including iodine deficiency, there has been a huge reduction in IDDs since the introduction of iodized salt in Uganda [37,45]. Given that all stakeholders consulted were residents of Northern Uganda, their concern was generally based on stakeholder observations in the field and further supports the necessity for agronomic biofortification for tackling IDDs in the specific region of Northern Uganda. Another outstanding opportunity was the *existence of fertile soil which is deficient in iodine* (O2). As agronomic biofortification is an agricultural-based intervention, the issue of land and soil fertility is crucial. The availability of iodine in the soil is regulated by geochemical processes involving the flow of iodine from oceans into inlands [10]. Food and Agriculture Organization (FAO) [39] noted that Uganda is largely mountainous which is an ecological disadvantage as it makes the level of iodine in soil low for plant intake. This supports the perception of the stakeholders when they point out the existence of iodine deficient soils as a key opportunity and could further support the need for agronomic iodine biofortification to increase iodine intake by crops. 

*Emerging fertilizer companies in Uganda* (O3) was only ranked relatively high by the agro-input group itself (Figure 2c) and, to a lesser extent, by the CDO group. Another point of deviation was existence of *government support and extension services* (O1), which was ranked highest by the CDO group and second highest by both the academic and NGO groups. This might be related to the fact that they often collaborate with governmental agencies when implementing community programs. In fact, the Community Development Officers (CDOs) are government employees directly involved in supervising interventions implemented in the community by both private and public agencies. The government of Uganda (through National Agricultural Research Organizations, NARO) has already demonstrated its support for the research and production of biofortified staples. While large research studies on the enhancement of vitamin A and iron concentrations in bananas are ongoing, with a release date expected in 2019 [56], iron beans and provitamin A orange sweet potatoes already exist and have reached, respectively, about 40,000 and 130,000 households in Uganda [57,58]. As such, there is already a history of governmental support for biofortification, which explains the high rankings of several stakeholder groups (CDO, academics and NGO). 

#### 3.2.3. Weaknesses

Despite holding a generally high level of positive perceptions about agronomic iodine biofortification (Figure 1), stakeholders expressed concerns over some factors that could negatively affect the implementation of this technology, especially in a Ugandan setting. The most crucial weaknesses of the technology include the following: *overuse of fertilizers causes toxicity* (W3) *and agronomic biofortification takes a longer time to supply iodine* (W4) (Figure 2b). The argument that agronomic biofortification is a longer route for providing iodine to humans has no scientific backing. In fact, agronomic biofortification is often considered a faster approach to enhancing the micronutrient contents of food crops when compared to genetic engineering and conventional plant breeding, which both take longer development periods [3]. Nevertheless, when compared to the consumption of micronutrient fortified food products or supplements which are developed in food processing or pharmaceutical industries, agronomic biofortification, which supplies iodine only after the crops have matured and are harvested, is a relatively longer approach. In regard to the fear that the overuse of fertilizer could cause toxicity (W3), the environmental impact of continual application of chemical fertilizers has been well studied. It is known that this leads to the accumulation of traces of heavy metals in soil which can be carried along food chains and cause poisoning, such as lead poisoning [59]. However, this depends on soil and plant characteristics as well as fertilizer application rates [60], which can be regulated.

Contradictory to other stakeholder groups, the CDO and NGO groups ranked *iodine is volatile and is easily lost after applying fertilizer* (W5) as the second most important weakness (Figure 2b). While this concern might be addressed by regulating the quantity of fertilizer applied and the application method, the volatility of iodine is a critical issue that needs to be taken into consideration during the design of the intervention. It has been shown that iodine is readily lost from the soil following its application [61]. The academic and agro-input groups also differed from other stakeholders when they produced relatively high priorities for weakness W1: *iodine fertilizer is not readily available* (Table 3)*.* This concern is particularly important given that biofortification through agronomic approaches is a relatively new intervention strategy for micronutrient deficiency. In many studies, micronutrient fertilizers have been formulated from laboratory chemicals mainly for experimental purposes and have been supplied in combination with carrier elements, e.g., potassium iodide and iodate [4,8,61] and Zn-enriched NPK fertilizers [62] have been used. To make these laboratory mineral fertilizer formulations available to the producers (e.g., farmers) at affordable prices, will require government effort to subsidize the cost of fertilizers as well as to regulate its importation and trade. Efforts by large international players who develop and disseminate biofortified staples could be complemented by national government efforts to facilitate the regulation of production and the import of mineral fertilizers as well as to subsidize their prices for the rural poor households. In Uganda, for example, HarvestPlus set up a successful collaboration with various national partners in order to provide iron biofortified beans and vitamin A biofortified sweet potatoes to, respectively, 39,000 and 132,000 households [58]. 

While the stakeholders noted that *fertilizers are expensive* (W2), it is rather surprising that the majority of the stakeholder groups consulted did not give a high score to this weakness. This might confirm the fact that the stakeholders generally agreed that the overall process of agronomic biofortification is a relatively inexpensive alternative (strength, S1) for controlling IDDs.

#### 3.2.4. Threats

In the case of threats, stakeholders are concerned about the *low knowledge and awareness* of the majority of Ugandan farmers about fertilizer application (T1) and the *general low fertilizer use in Uganda* (T3) (Figure 2d). Empirical evidence underlines the low use of fertilizers in Uganda, mainly because of high costs of fertilizers and low knowledge of farmers on their application [63,64,65]. These aspects increase the need for awareness creation and training of farmers on the (benefits of) application of fertilizers. As opposed to the CDO group, all other stakeholder groups scored highly on threat T5 (*farmers have competing needs for increase in yield other than quality/nutrients*). The agro-input group gave a priority score of up to 0.471 (47.1%) to this threat factor (T5), followed by the academic group (0.301), NGO group (0.297) and government extension officers (0.253) groups (Table 3). The concern that farmers might have competing needs for improvement in yield (quantity) over quality (nutrient) of production is expected as many farmers in Uganda are subsistent in their operation and would like to produce a greater quantity of food to feed their households and have a surplus to generate income [38]. However, agronomic biofortification does not negatively affect the yield of crops [66]. In fact, biofortification generally seeks to improve the micronutrient density of crop varieties which already have desired production and consumption attributes, such as high yielding varieties [66]. As such, this concern can be addressed through farmer education and training. 

## 4. Conclusions and Recommendations

This study offers the first application of SWOT-AHP technique in appraising factors that could influence the implementation of a micronutrient intervention, namely, agronomic iodine biofortification. The study consulted key stakeholders who influence the production, regulatory and market environments for agricultural and novel innovations. We have shown that stakeholders are receptive and optimistic about agronomic iodine biofortification and provided key issues that need to be addressed to successfully promote the intervention in Uganda and elsewhere. 

In summary, the technology is taken as culturally acceptable in Uganda with the potential to reduce chronic disease risk. However, stakeholders also understood agronomic biofortification to be less expensive, simple and easily to implement. These aspects should be taken into account when designing the intervention to ensure wide spread adoption. For instance, affordable packs of iodine fertilizers with clear instructions on application could be designed to ensure that farmers in a developing country setting can easily understand and afford it. This will also handle a key threat envisaged mainly by the farmers’ representatives, who expressed fear over low knowledge and awareness of Ugandan farmers on fertilizer use. 

In terms of policy implications, the perceived and empirical evidence of low (awareness and knowledge of) fertilizer use by farmers further lend support for farmers’ sensitization and training to embrace and improve (iodine) fertilizer use. This should be integrated into already existing government extension service systems—a key opportunity according to the stakeholders. However, given that most farmers in Uganda are subsistent in their operation and only few can afford fertilizers on a regular basis, as noted by the stakeholders, the government could also consider measures to subsidize the iodine fertilizers for farmers once available. In addition, support is also needed to improve the development and/or import of micronutrient fertilizers, especially iodine fertilizers, as they do not currently exist on a commercial scale in Uganda and many other countries. This will allay the fear of iodine fertilizers being unavailable in Uganda. 

One critical fear of the stakeholders is the fact that iodine is volatile and could be lost from the soil or plant upon application. This offers a research and development need that the producers of iodine fertilizers should take in account when formulating iodine fertilizers. This might be handled by careful selection of carrier elements or compounds for the iodine fertilizers. Stakeholders are also concerned about the likelihood of farmers choosing improvement in yield over improvement in nutrient quality. As such, agronomic iodine biofortification should target crop varieties that are already known to be high yielding and are desired by farmers. 

The stakeholders consulted also pointed out that there are visible goiter cases in the community, though there are have been recent studies on prevalence of IDDs. On this basis, we recommend that future research could investigate the prevalence of IDDs in Uganda to further inform proper planning for an intervention. As the findings from the current study are based on perceptions of key stakeholder groups, future research could aim to provide empirical evidence for the perceived factors that are considered key for developing agronomic iodine biofortification. Thereby, one could also identify and examine potential solutions to counter the effect of some of the negative (weakness and threat) factors generated and considered important for the development and implementation of agronomic iodine biofortification. 

It is important to note that iodine biofortification, i.e., the case of our SWOT-AHP study, is currently not implemented as a policy intervention in Uganda. If it were to be introduced in the future, it would be important to monitor changes in stakeholder perceptions over time, e.g., through a before–after SWOT-AHP design. Future research is also needed to further evaluate the application of this method by examining its value in different contexts, e.g., targeting other countries, regions, nutrients or crops, before or after introduction. Furthermore, while this and similar stakeholder analyses have focused on a single intervention [33,34], aimed at identifying and evaluating important factors to consider when designing and implementing a particular intervention, AHP can also be used to compare multiple interventions. In the case of (iodine) biofortification, one could opt to compare the findings with stakeholder perceptions on existing interventions (e.g., fortification, supplementation, biofortification). However, one has to bear in mind that biofortification should be considered a complementary micronutrient strategy, as, in reality, a combination of interventions is often required to tackle malnutrition.

Given the aforementioned lack of stakeholder analysis in this field, our study, which builds upon the SWOT-AHP method, illustrates the potential value of this method in order to identify, prioritize and compare perceptions of various types of stakeholders. This is crucial, as the success of implementing iodine biofortification in the future will require a joint effort of academics and researchers working to develop appropriate iodine biofortification packages; the government, regulating the production and marketing environment; and government and NGO extension officers, agro-input companies and community development officers offering the necessary training to farmers and creating awareness in the community. As such, our results can provide a basis for researchers aiming to conduct stakeholder analyses in the field of agri-food based (health) interventions and biofortification, in particular, in Africa or beyond. 

## Figures and Tables

**Table 1:** An example of a pairwise comparison table (Analytical Hierarchy Process (AHP) method).

Increasing Importance									Equal Importance	Increasing Importance								
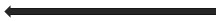										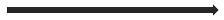								
	9	8	7	6	5	4	3	2	1	2	3	4	5	6	7	8	9	
Strength 1																		Strength 2
Strength 1																		Strength 3
Strength 2																		Strength 3

**Table 2:** Key Strengths, Weaknesses, Opportunities and Threats (SWOT) factors as perceived by all stakeholders in the focus group.

Strengths	Weaknesses
* Overall inexpensive approach for fighting iodine deficiency. * The technology is relatively simple and can be easily implemented by farmers. * Agronomic biofortification is culturally acceptable in Uganda. * Effective in reducing chronic disease risk. * Iodine fertilizers can be blended with other plant nutrients. Iodized products can offer other nutrients. - It addresses both food and health (nutrition) security. - It adds value to existing crops.	* Iodine fertilizers are not readily available. * Fertilizers are generally expensive and not affordable by most farmers in Uganda. * Iodine is highly volatile and can easily be lost after being applied to the soil or plants. * Continuous use of (iodine) fertilizers can accumulate in the soil and become toxic. * It takes a longer time for agronomic biofortification to supply iodine (as compared to, e.g., fortified foods).
**Opportunities**	**Threats**
* Government support and extension services for innovative agricultural technologies. * Increasing number of fertilizer companies in Uganda. * Existence of fertile soils that are deficient in iodine. - Does not increase salt intake, as compared to iodized salt. * Fertilizer policy exists in Uganda. Other types of fertilizers are already on the market. * High prevalence of iodine deficiency disorders, visible goiter patients are reported in society. - Existence of staple crops that are deficient in iodine. - Farmers in Northern Uganda have abundant land for the production of biofortified crops. - Government and international agencies are willing to fund agricultural development initiatives in North Uganda.	Ongoing campaigns to lower the use of synthetic fertilizers and become organic. * Limited knowledge and awareness of farmers on fertilizer application. * Fertilizer application is negatively affected by environmental factors, such as soil (e.g., organic matter, pH (acidity), texture) and weather. - High tendency of product counterfeiting in Uganda that will affect acceptance of biofortified products with no visible features. * Misconception of technology (e.g., agronomic biofortification versus GM technology). * There is generally low fertilizer use by farmers in Uganda. * Farmers have competing needs for increase in yield rather than quality of farm produce. - General negative attitude of people in the society on new products and technologies.

* The five most important factors (in each SWOT category) that were selected to be used in pairwise comparisons (AHP) are shown with asterisks. GM, Genetic Modification

**Table 3:** SWOT-AHP priority scores for factors that could influence the development of agronomic iodine biofortification.

SWOT Categories and Factors	Local Priority Scores (Within Factors)						Global Priority Scores (Across Factors)						
	ACAD	NGO	GOV. ExT	CDO	AGRO. INPUT	FARM	ACAD	NGO	GOV. ExT	CDO	AGRO. INPUT	FARM	Overall
Strengths							**0.341**	**0.546**	**0.308**	**0.241**	**0.323**	**0.143**	**0.317**
S1: Cheap way for fighting IDDs	0.123	0.219	0.134	0.093	0.077	0.059	0.042	0.120	0.041	0.022	0.025	0.008	0.043
S2: Simple, easily to implement	0.194	0.045	0.041	0.062	0.291	0.339	0.066	0.025	0.013	0.015	0.094	0.048	0.044
S3: Culturally acceptable in Uganda	0.304	0.042	0.140	0.372	0.429	0.060	0.104	0.023	0.043	0.090	0.139	0.009	0.068
S4: Reduce risks for chronic diseases	0.317	0.231	0.485	0.340	0.062	0.212	0.108	0.126	0.149	0.082	0.020	0.030	0.086
S5: Can be blended with other nutrients	0.062	0.464	0.200	0.133	0.142	0.330	0.021	0.253	0.062	0.032	0.046	0.047	0.077
Weaknesses							**0.074**	**0.059**	**0.051**	**0.058**	**0.052**	**0.076**	**0.062**
W1: Iodine fertilizers not readily available	0.327	0.038	0.069	0.065	0.283	0.051	0.024	0.002	0.004	0.004	0.015	0.004	0.009
W2: Fertilizers are expensive	0.231	0.056	0.131	0.100	0.103	0.052	0.017	0.003	0.007	0.006	0.005	0.004	0.007
W3: Overuse of fertilizers causes toxicity	0.069	0.128	0.279	0.429	0.401	0.426	0.005	0.008	0.014	0.025	0.021	0.032	0.018
W4: Takes a long time to supply iodine	0.313	0.471	0.448	0.169	0.107	0.259	0.023	0.028	0.023	0.010	0.006	0.020	0.018
W5: Iodine is volatile and can be lost	0.059	0.306	0.073	0.237	0.107	0.213	0.004	0.018	0.004	0.014	0.006	0.016	0.010
Opportunities							**0.408**	**0.329**	**0.546**	**0.552**	**0.574**	**0.584**	**0.499**
O1: Government support and extension	0.262	0.244	0.082	0.305	0.077	0.104	0.107	0.080	0.045	0.168	0.044	0.061	0.084
O2: Existence of fertile soils, deficient in iodine	0.156	0.075	0.390	0.033	0.185	0.370	0.064	0.025	0.213	0.018	0.106	0.216	0.107
O3: Emerging fertilizer companies	0.098	0.047	0.069	0.290	0.241	0.070	0.040	0.015	0.038	0.160	0.138	0.041	0.072
O4: High prevalence of IDDs	0.342	0.529	0.388	0.067	0.436	0.397	0.140	0.174	0.212	0.037	0.250	0.232	0.174
O5: Fertilizer policy in Uganda	0.141	0.104	0.072	0.305	0.060	0.059	0.058	0.034	0.039	0.168	0.034	0.034	0.061
Threats							**0.176**	**0.067**	**0.095**	**0.149**	**0.052**	**0.198**	**0.123**
T1: Low knowledge and awareness of farmers	0.187	0.247	0.140	0.405	0.234	0.571	0.033	0.017	0.013	0.060	0.012	0.113	0.041
T2: Fertilization affected by environment	0.053	0.064	0.071	0.062	0.124	0.062	0.009	0.004	0.007	0.009	0.006	0.012	0.008
T3: Low fertilizer use in Uganda	0.311	0.286	0.457	0.299	0.124	0.130	0.055	0.019	0.043	0.045	0.006	0.026	0.032
T4: Likely misconception of technology	0.147	0.106	0.079	0.054	0.049	0.095	0.026	0.007	0.008	0.008	0.003	0.019	0.012
T5: Competing needs for yield	0.301	0.297	0.253	0.180	0.471	0.142	0.053	0.020	0.024	0.027	0.024	0.028	0.029

ACAD: academic group; NGO, Non-Governmental Organization; GOV.EXT: government extension group; CDO: Community Development Officers; AGRO.INPUT: agro-input company sample; FARM: elite farmers group. IDDs, Iodine Deficiency Disorders. Note: **Bold** figures indicate SWOT group scaling or priority scores.

## Supplementary Materials

The following are available online at http://www.mdpi.com/2072-6643/10/4/407/s1, Figure S1: Global priority scores of the academic group, Figure S2: Global priority scores of NGO representatives, Figure S3: Global priority scores of government extension representatives, Figure S4: Global priority scores of CDO, Figure S5: Global priority scores of agro-input companies, Figure S6: Global priority scores of the elite farmers’ group.
