# Supplementary material for: Stakeholders’ Perceptions of Agronomic Iodine Biofortification: A SWOT-AHP Analysis in Northern Uganda
Source: Nutrients. 2018 Mar 24;10(4):407. doi: 10.3390/nu10040407 (PMC5946192; doi:10.3390/nu10040407)
Supplement: Supplementary file 1 [file nutrients-10-00407-s001.pdf]

Appendix 1. Global Priority Scores, per stakeholder group

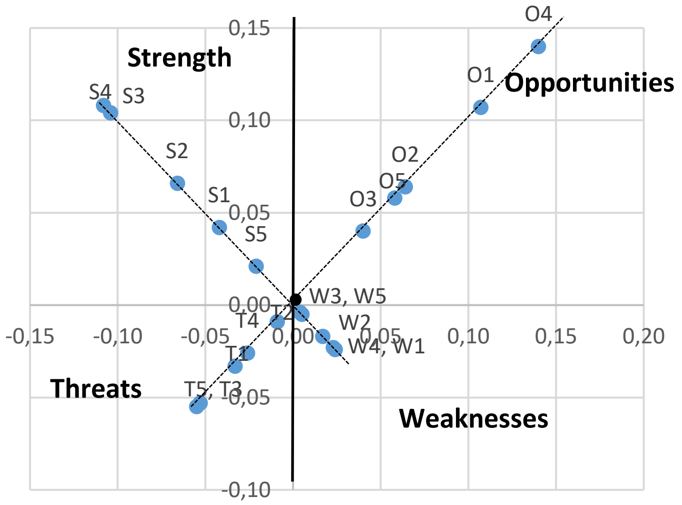

Fig 1: Global priority scores of the academic group

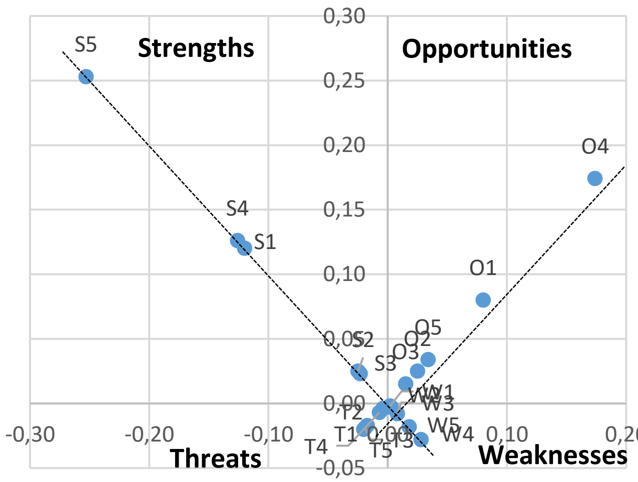

Fig 2: Global priority scores of NGO representatives

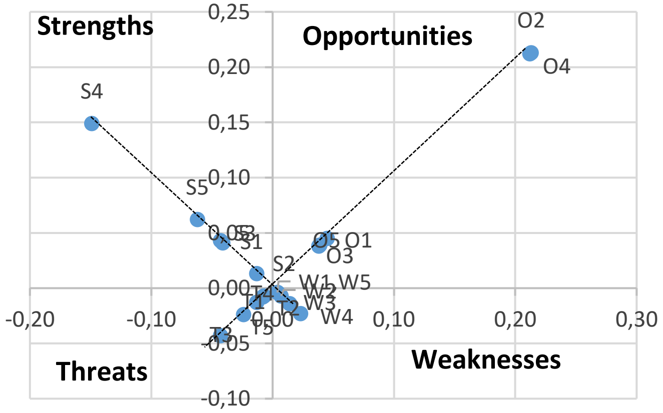

Fig 3. Global priority scores of government extension representatives

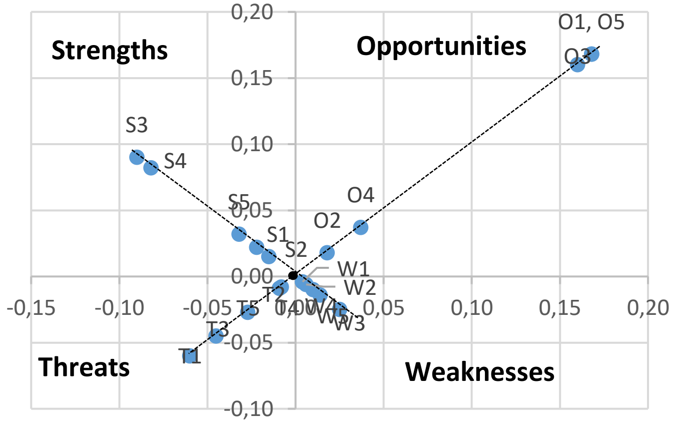

Fig 4. Global priority scores of CDO

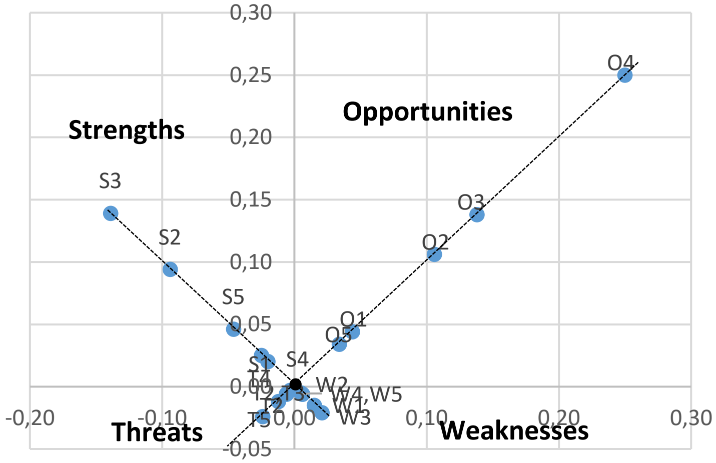

Fig 5. Global priority scores of agro-input companies

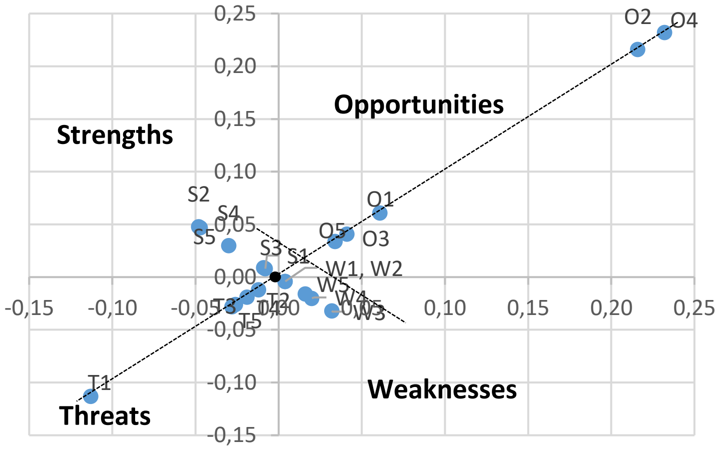

Fig 6. Global priority scores of the elite farmers' group
